# Performance Evaluation of a Nanofluid-Based Direct Absorption Solar Collector with Parabolic Trough Concentrator

**DOI:** 10.3390/nano5042131

**Published:** 2015-12-04

**Authors:** Guoying Xu, Wei Chen, Shiming Deng, Xiaosong Zhang, Sainan Zhao

**Affiliations:** 1 School of Energy and Environment, Southeast University, 210096 Nanjing, China; E-Mails: agdmwdg@163.com (W.C.); rachpe@seu.edu.cn (X.Z.); 15651693509@163.com (S.Z.); 2 Department of Building Services Engineering, The Hong Kong Polytechnic University, Hong Kong; E-Mail: simon.shi-ming.deng@polyu.edu.hk

**Keywords:** solar collector, nanofluid, temperature distribution, collection efficiency

## Abstract

Application of solar collectors for hot water supply, space heating, and cooling plays a significant role in reducing building energy consumption. For conventional solar collectors, solar radiation is absorbed by spectral selective coating on the collectors’ tube/plate wall. The poor durability of the coating can lead to an increased manufacturing cost and unreliability for a solar collector operated at a higher temperature. Therefore, a novel nanofluid-based direct absorption solar collector (NDASC) employing uncoated collector tubes has been proposed, and its operating characteristics for medium-temperature solar collection were theoretically and experimentally studied in this paper. CuO/oil nanofluid was prepared and used as working fluid of the NDASC. The heat-transfer mechanism of the NDASC with parabolic trough concentrator was theoretically evaluated and compared with a conventional indirect absorption solar collector (IASC). The theoretical analysis results suggested that the fluid’s temperature distribution in the NDASC was much more uniform than that in the IASC, and an enhanced collection efficiency could be achieved for the NDASC operated within a preferred working temperature range. To demonstrate the feasibility of the proposed NDASC, experimental performances of an NDASC and an IASC with the same parabolic trough concentrator were furthermore evaluated and comparatively discussed.

## 1. Introduction

Solar energy utilization is one of the effective ways to relieve the pressures on energy shortage and environmental conservation. At present, solar heating technology is widely employed for domestic hot water supply in buildings. On a global scale, the total heating capacity based on solar collector was estimated at 330 GW by the end of 2013 [1]. However, compared with the extensive low-temperature solar energy utilization (below 80 °C), for example, the domestic hot water supply, medium-temperature (80–250 °C) solar energy utilization, such as solar thermal refrigeration and air conditioning, solar thermal power generation, as well as solar energy based industrial process heating, is still relatively limited, and hence has great potential for growth. Therefore, further studies and developments for medium-temperature solar collectors are necessary.

For commonly used solar collectors, solar radiation is firstly captured and absorbed by spectral selective coating on the collectors’ tube/plate wall, converted into heat, and then indirectly transferred to the heat-transfer fluid (HTF) inside the tubes. Unlike these commonly used indirect absorption solar collectors (IASC), novel nanofluid-based direct absorption solar collectors (NDASC) have been recently proposed [2,3,4,5,6,7,8,9,10,11,12,13,14,15]. An NDASC employs uncoated collector tube/plate in which nanofluid acts as both a solar absorbing medium and heat-transfer fluid. Nanofluid, a kind of stable mixture of the fluid and nanoparticles, could exhibit strong spectral absorption properties and improve thermo-physical properties due to the nano-scale size particles dispersed in a heat-transfer fluid [2,3,4,5,6]. Until now, studies on NDASC have mostly focused on evaluating the optical properties of water or ethylene glycol suspension added with various nanoparticles, and performances for low-temperature solar collectors [7,8,9,10,11,12]. Karami *et al.* [7] prepared carbon nanotubes/water nanofluid and investigated its performances when used in low-temperature solar collectors. Sani *et al.* [8] reported the optical characterization of single-wall carbon nanohorns/ethylene glycol for solar absorbing applications. Otanicar *et al.* [9] experimentally investigated a flat-plate solar collector using nanofluid made from water added with a variety of nanoparticles including carbon nanotubes, graphite, and silver, and the study results suggested that the efficiency could be improved by up to 5%. Tyagi *et al.* [10] established a two-dimensional heat transfer mathematical model of a flat-plate NDASC using aluminum/water nanofluid.

Previous studies demonstrated that the use of NDASCs could lead to an acceptable efficiency with a reduced manufacturing cost for low-temperature solar collectors. Such an advantage may also make much sense to a medium-temperature solar collector. This is because the stability and durability of the coating used in conventional medium-temperature solar collectors decrease at a higher temperature, leading to a significantly increased manufacturing and maintenance cost. However, the studies on NDASCs for medium-temperature applications are limited. de Risi. *et al.* [16] simulated the performances of a parabolic trough collector using a transparent receiver tube combined with gas-based nanofluids, for high-temperature (650 °C) solar collection. Taylor *et al.* [17] theoretically analyzed a type of power tower nanofluid-based solar receiver with a working temperature of 270 °C, and suggested that the potential of nanofluid enhancement in efficiency was up to 10% as compared to surface-based solar receiver. However, solar radiation intensity incident on to the collector plate was assumed to be uniform in these studies.

**Figure 1:**
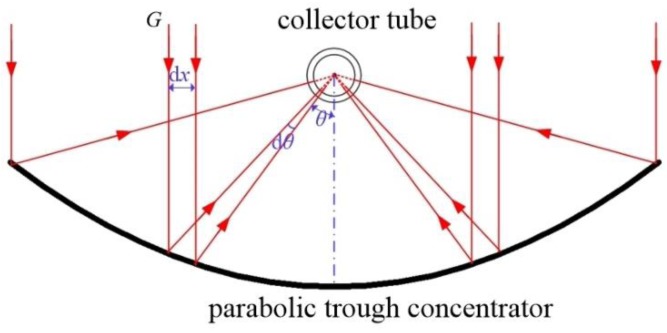
Schematic diagram of the cross section of a parabolic trough concentrating solar collector.

Figure 1 shows a typical parabolic trough concentrating solar collector for medium-temperature solar heat collection. It is mainly composed of a collector tube and a parabolic trough concentrator [18]. The incident solar radiation is reflected to the collector tube placed in the focal line of the concentrator, and then heats the heat transfer fluid (HTF). Heat transfer oil is commonly used as working fluid in a medium-temperature solar collector, because of its higher vaporization temperature at above 300 °C. The solar radiation intensity along the tube circumference is extremely non-uniform, resulting in a more complicated heat transfer mechanism inside tube. There is however no existing literature reporting the operating characteristics of an NDASC under solar-concentrating medium-temperature condition, and taking the non-uniform incident solar radiation into account.

Therefore, this paper reports on an investigation of the operating characteristics of a proposed novel NDASC under solar-concentrating medium-temperature operating conditions. Firstly, the detailed heat transfer mechanism of the NDASC with parabolic trough concentrator is theoretically evaluated. This is followed by a theoretical analysis on the operating characteristics of both the NDASC and a conventional IASC. Finally, the performances of an NDASC and an IASC with the same parabolic trough concentrator are experimentally evaluated, to demonstrate the feasibility of the proposed NDASC.

## 2. Results and Discussion

### 2.1. Solar Collection Principles and Theoretical Evaluation of Heat Transfer

#### 2.1.1. Solar Collection Principles

In a concentrating medium-temperature solar collector, its concentrator reflects the solar radiation to its collector tube, so that the received solar radiation can be improved by several folds.

**Figure 2:**
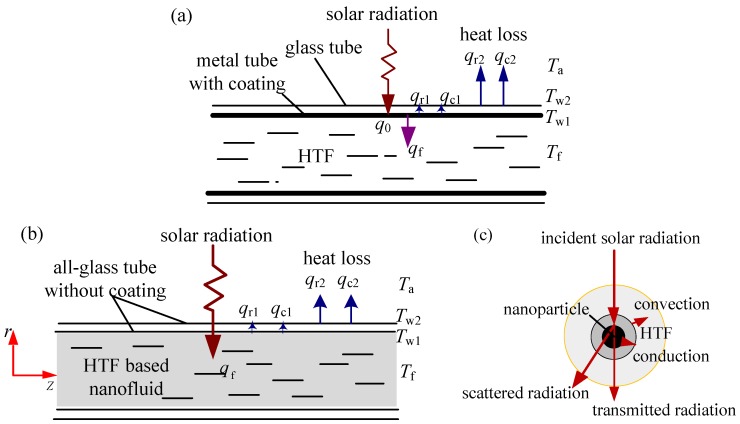
Schematics of solar collection principles. (**a**) A conventional indirect absorption solar collector (IASC); (**b**) The proposed novel nanofluid-based direct absorption solar collector (NDASC); (**c**) The heat transfer around nanoparticles inside the tube of NDASC.

The solar collection principle of a conventional IASC is shown in Figure 2a. The most commonly used medium-temperature collector tube is made of metal-glass evacuated tube. A metal tube whose surface coated with selective absorbing film is coaxially placed inside a glass tube. The space between the metal and glass tube is vacuumized to reduce the heat loss. The incident solar radiation is firstly captured by the coatings, converted into heat, and then transferred to heat-transfer fluid (HTF). Meanwhile, a part of the absorbed heat is lost to ambient air through the outer tube surface.

By contrast, the proposed NDASC employs an all-glass evacuated tube without spectral selective coating. As shown in Figure 2b, a kind of HTF added with certain nanoparticles (CuO in this study) acts as working fluid. The nanoparticles dispersed in the HTF inside the inner tube directly capture the solar radiation instead of the tube wall coating. For a specific nanoparticle, its heat transfer process is described in Figure 2c. Apart from the transmitted and scattered radiation, the incident radiation is absorbed by the nanoparticles, converted into heat and then transferred to the surrounding HTF. Meanwhile, a very small proportion of the incident radiation is absorbed by the HTF (synthetic oil in this study), as the synthetic oil mainly absorbs infrared radiation.

#### 2.1.2. Theoretical Evaluation of Heat Transfer in the Novel NDASC

**Figure 3:**
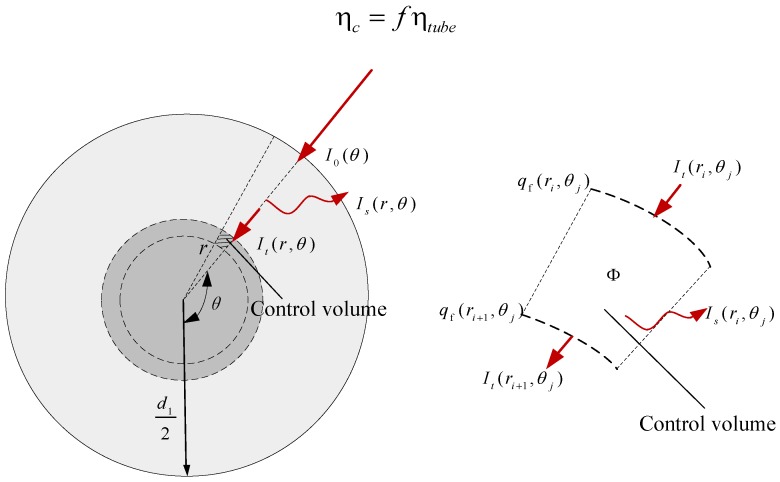
The solar radiation transfer inside the tube of the NDASC.

Figure 3 shows the solar radiation transfer inside the all-glass evacuated tube of the NDASC. The transmitted radiation intensity, *I_t_*, within the nanofluid inside the tube is assumed to vary in only two dimensions (*r*-direction and θ-direction in a cylindrical coordinate). Therefore, there is no change of *I*_t_ along *z*-direction. According to the Beer-Lambert Law, the radiation intensity distribution within the collector tube can be obtained by using a radiative transfer equation as follows:

(1) $$\frac{\partial I_{t}(r,\theta )}{\partial r}=-K_{e}I_{t}(r,\theta )$$

where *K*_e_ is the extinction coefficient of the nanofluid, m^−1^; $I_{t}(r,\theta )$ the transmitted radiation intensity at coordinate (*r*, θ).

Given the initial incident intensity around the cylindrical surface of tube, $I_{0}(\theta )$, the transmitted radiation intensity at radius *r* can be calculated by:

(2) $$I_{t}(r,\theta )=e^{-K_{e}r}I_{0}(\theta )$$

Apart from the transmitted radiation $I_{t}(r,\theta )$, there is some radiation scattered outside from the nanofluid as scattered radiation, $I_{s}(r,\theta )$. The absorption coefficient of the nanofluid, *K*_a_ is defined as follows:

(3) $$\frac{I_{t}(r,\theta )+I_{s}(r,\theta )}{I_{0}(\theta )}=e^{-K_{a}r}$$

Hence, the total absorbed heat flux of the nanofluid from the cylindrical surface to the radius *r*, $q_{\text{f}}(r,\theta )$, is calculated by using absorption coefficient of the nanofluid:

(4) $$q_{\text{f}}(r,\theta )=I_{0}(\theta )-[I_{t}(r,\theta )+I_{s}(r,\theta )]=I_{0}(\theta )(1-e^{-K_{a}r})$$

The solar radiation absorbed by the nanofluid to increase its temperature, is assigned as an internal heat source. Take a specific control volume of nanofluid, *dA*, shown in Figure 3 as an example, the internal heat source intensity, Φ, is calculated by:

(5) $$\Phi =\frac{\delta [q_{\text{f}}(r,\theta )rd\theta ]}{dA}=\frac{q_{\text{f}}(r_{i+1},\theta_{j})r_{i+1}-q_{\text{f}}(r_{i},\theta_{j})r_{i}}{({r_{i}}^{2}-{r_{i+1}}^{2})/2}$$

Therefore, the heat-transfer inside the collector tube of the NDASC can be mathematically described by a heat-transfer equation with an internal heat source within the nanofluid, as follows:

(6) $$\frac{\partial (\rho T_{f})}{\partial t}+\frac{\partial (\rho u_{x}T_{f})}{\partial x}+\frac{\partial (\rho u_{y}T_{f})}{\partial y}+\frac{\partial (\rho u_{z}T_{f})}{\partial y}=\frac{\partial }{\partial x}[\frac{k}{C}\frac{\partial T_{f}}{\partial x}]+\frac{\partial }{\partial y}[\frac{k}{C}\frac{\partial T_{f}}{\partial y}]+\frac{\partial }{\partial z}[\frac{k}{C}\frac{\partial T_{f}}{\partial z}]+\Phi$$

where *T*_f_ is the temperature of the working fluid, K; ρ the density of working fluid, kg/m^3^; *C* the specific heat of the working fluid, J/kg·K; *k* the thermal conductivity of working fluid, W/(m·K); *u_x_*, *u_y_*, *u_z_* the velocity of working fluid in the *x*-direction, *y*-direction, and *z*-direction respectively, m/s; *t* the time.

When the tube wall temperature is increased to be higher than ambient air, part of the absorbed heat is firstly transferred from the inner tube to the outer tube, and finally to ambient air. The heat transfer between the inner and outer tube includes a radiation part and a convection part. Since the wall temperature varies along with the tube circumference and tube length, the heat flux between the inner and outer tube due to radiation (*q_r_*_1_) at coordinate $\left(\frac{d_{1}}{2},\theta ,z\right)$, can be evaluated by Equation (7). Furthermore, *q_r_*_1_ can be completely absorbed by the outer tube because it is infrared radiation.

(7) $$q_{r1}=\frac{\sigma \left[{T_{w1}}^{4}(\theta ,z)-{T_{w2}}^{4}(\theta ,z)\right]}{\frac{1}{\varepsilon_{1}}+\frac{d_{1}}{d_{2}}\left(\frac{1}{\varepsilon_{2}}-1\right)}$$

where σ is the Stefan-Boltzmann constant, 5.67 × 10^−8^ W/(m^2^·K^4^); $T_{w1}(\theta ,z)$, $T_{w2}(\theta ,z)$ the wall temperature of the inner and outer tube, respectively at coordinate (θ,*z*); *d*_1_, *d*_2_ the diameter of the inner and outer tube, respectively; ε_1_, ε_2_ the surface emissivity of the inner and outer tube, respectively.

As there is air in the space between the inner and outer tubes, the heat flux through the air due to convection, *q*_c1_, is evaluated by:

(8) $$q_{c1}=h_{c1}\left[T_{w1}(\theta ,z)-T_{w2}(\theta ,z)\right]$$

where the convective heat transfer coefficient, *h_c_*_1_, is calculated according to reference [19].

The heat flux due to thermal radiation loss, *q*_r2_, and that due to convection loss, *q*_c2_, from the outer tube to ambient air, can be respectively calculated as:

(9) $$q_{r2}=\sigma \varepsilon_{2}\left[{T_{w2}}^{4}(\theta ,z)-{T_{a}}^{4}\right]$$

(10) $$q_{c2}=h_{c2}[T_{w2}(\theta ,z)-T_{a}]$$

The convection heat transfer coefficient, *h_c_*_2_, is calculated according to reference [19] as:

(11) $$h_{c2}=0.0239\frac{k_{\text{a}}}{d_{2}}{\left(\frac{\rho_{\text{a}}V_{\text{a}}\cdot d_{2}}{\mu_{\text{a}}}\right)}^{0.805}$$

where *T_a_* is ambient air temperature, K; *k*_a_ the coefficient of thermal conductivity of air, W/(m·K); *V*_a_ the ambient wind velocity, m/s; ρ_a_ air density, kg/m^3^; μ_a_ air viscosity, Pa·s.

According to energy balance, the heat loss from the inner tube to the outer tube equals to that from the outer tube to ambient air, which is expressed as:

(12) $$\frac{d_{1}}{2}(\int_{-\pi }^{\pi }q_{\text{r1}}d\theta +\int_{-\pi }^{\pi }q_{\text{c1}}d\theta )=\frac{d_{2}}{2}(\int_{-\pi }^{\pi }q_{\text{r2}}d\theta +\int_{-\pi }^{\pi }q_{\text{c2}}d\theta )$$

The solar collection efficiency of the collector tube is the heat energy gain of working fluid divided by the total solar radiation incident onto the collector tube surface:

(13) $$\eta_{tube}=\frac{\int_{-\pi }^{\pi }d\theta \int_{0}^{\frac{d_{1}}{2}}\rho_{C}u_{\text{f,o}}(r,\theta )\left[T_{\text{f,o}}(r,\theta )-T_{\text{f,i}}\right]rdr}{\frac{d_{1}}{2}L\int_{-\pi }^{\pi }I_{0}(\theta )d\theta }$$

where, $T_{\text{f,o}}(r,\theta )$ is outlet temperature of fluid at coordinate position (r,θ,z=L); $u_{\text{f,o}}(r,\theta )$ the velocity of fluid at coordinate position (r,θ,z=L); *L* the collector tube length; $T_{\text{f,i}}$ the inlet temperature of fluid.

For a concentrating solar collector, the influences of the concentrator’s assembly error, sun tracking error, as well as mirror stains on solar collection efficiency should be taken into account. Hence, an optical efficiency factor of concentrator, *f*, is introduced to represent the above influences [19,20]. The solar collection efficiency of the concentrating solar collector, $\eta_{c}$, is then evaluated by:

(14) $$\eta_{c}=f\eta_{tube}$$

#### 2.1.3. Theoretical Evaluation of Heat Transfer in an IASC

Theoretical calculation of an IASC has also been carried out for the purpose of performance comparison. Compared with a NDASC, the differences include that the working fluid in an IASC is HTF rather than nanofluid, and that the solar radiation is absorbed by the coating on the inner tube wall, as shown in Figure 2a. Hence, the heat transfer inside the IASC tube can be mathematically described by:

(15) $$\frac{\partial (\rho T_{f})}{\partial t}+\frac{\partial (\rho u_{x}T_{f})}{\partial x}+\frac{\partial (\rho u_{y}T_{f})}{\partial y}+\frac{\partial (\rho u_{z}T_{f})}{\partial z}=\frac{\partial }{\partial x}[\frac{k}{C}\frac{\partial T_{f}}{\partial x}]+\frac{\partial }{\partial y}[\frac{k}{C}\frac{\partial T_{f}}{\partial y}]+\frac{\partial }{\partial z}[\frac{k}{C}\frac{\partial T_{f}}{\partial z}]$$

As seen, for Equation (15), unlike Equation (6) for an NDASC, there is no internal heat source involved in the equation.

The absorbed heat flux around the tube wall surface, $q_{0}(\theta )$, is calculated by using the overall spectral absorptivity of the coating, α, as:

(16) $$q_{0}(\theta )=\alpha I_{0}(\theta )$$

Furthermore, the calculation method of heat loss and collection efficiency for the IASC are the same as that for the NDASC, *i.e.*, Equations (7)–(14), shown in Section 2.1.2.

### 2.2. Properties of CuO/Oil Nanofluid

**Figure 4:**
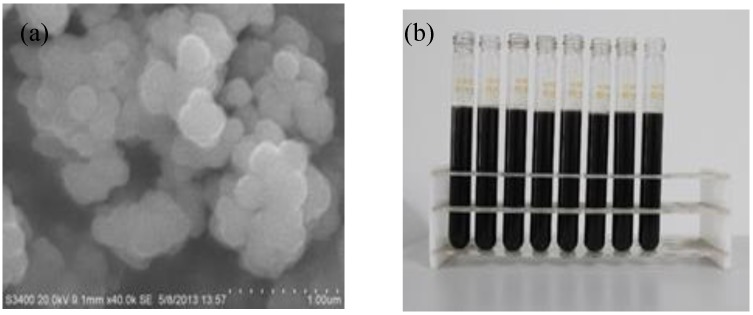
(**a**) Scanning Electron Microscope (SEM) image of CuO nanoparticles; (**b**) Photos prepared CuO/oil nanofluid used in the NDASC.

In the current study, the CuO/oil nanofluid was prepared and used as working fluid for the NDASC in this study. The base liquid of the nanofluid was WD type synthetic oil. Figure 4a shows a Scanning Electron Microscope (SEM) image of the used spherical CuO nanoparticles having the size of 200 nm. The specific surface area of nanoparticles was 80 m^2^/g. Figure 4b shows the laboratory prepared CuO/oil nanofluid. The mass fraction of the added CuO nanoparticles in the synthetic oil was 0.055 wt %. The physical properties of the CuO nanoparticles, synthetic oil, and the prepared CuO/oil nanofluid are listed in Table 1.

**Table 1:** Physical properties of the synthetic oil and nanofluid.

Parameters	*C_p_*	ρ	*k*	μ	*K*_e_	*K*_a_
Unit	J·(kg·K)^−1^	kg·m^−3^	W·(m·K)^−1^	Pa·s	m^−1^	m^−1^
CuO nanoparticles	475	6300	33	-	-	-
synthetic oil	2.14 × 10^3^	814.7	0.12	6.2 × 10^−3^	2	2
0.055 wt % CuO/oil nanofluid	2.139 × 10^3^	815.1	0.1201	6.2 × 10^−3^	514	103

Note: Listed values are the physical parameters at a temperature of 100 °C. Data of CuO nanoparticles and synthetic oil were obtained from manufacturers’ product manuals.

To investigate the solar absorption characteristics of the nanofluid, light transmissivity of the prepared nanofluid was measured by using a spectrophotometer with an integrating sphere. Consequently, its extinction coefficient $K_{\text{e}}(\lambda )$ and absorption coefficient $K_{\text{a}}(\lambda )$ at solar radiation wavelength range was obtained, respectively.

**Figure 5:**
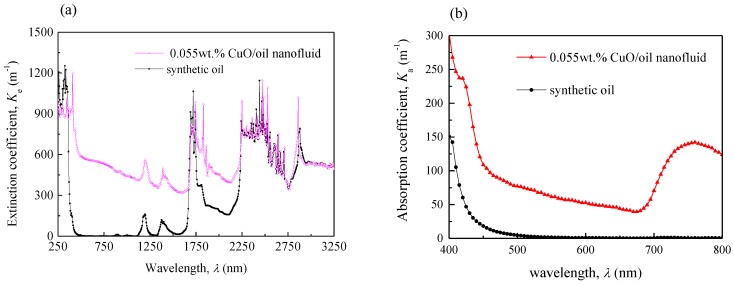
Extinction and absorption spectra of CuO/oil nanofluid and pure synthetic oil. (**a**) Variations of extinction coefficient with wavelength; (**b**) Variations of absorption coefficient with wavelength.

Figure 5 shows the extinction coefficient and absorption coefficient of both the nanofluid and pure synthetic oil. As seen, it is clear that adding CuO nanoparticles significantly improved the extinction and absorption coefficient of a fluid. In particular, in the range of the visible light wavelength, as shown in Figure 5b, the averaged absorption coefficient of synthetic oil was less than 2.0. By contrast, the averaged *K*_e_ of the CuO/oil nanofluid among the solar radiation spectrum (250–3300 nm wavelength) range reached 514 and *K*_a_ reached 103. The value of *K*_a_ was smaller than that of *K*_e_, because a part of the incident solar radiation was scattered from the nanofluid.

According to Equations (2) and (4), when the incident solar radiation goes through nanofluid inside a collector tube with diameter of 0.045 m, the ratio of the transmitted solar radiation to the initial incident solar radiation is $e^{-514\times 0.045}\text{=}9\times 10^{-11}$. Apart from the scatted radiation, the ratio of the absorbed solar radiation to the initial incident solar radiation equals to $(\text{1-}e^{-103\times 0.045})\text{=}0.99$, demonstrating that almost all the incident solar radiation is absorbed within the solar collector tube.

### 2.3. Theoretical Analysis on the Characteristics of the NDASC and the IASC.

#### 2.3.1. Calculation Conditions

Using the theoretical calculation equations presented in Section 2.1, and the properties of synthetic oil and CuO/oil nanofluid listed in Table 1, solar collection characteristics for both the NDASC and the IASC were numerically evaluated under different working temperature conditions, by using the UDF (User-Defined Function) of a commercial CFD software package. In the evaluation, the design parameters of the NDASC and the IASC listed in Table 2 were used. The parabolic trough solar concentrators used in both two collectors were the same, with geometric concentrating ratio (Wπd1) of 7.36. For the NDASC, CuO/oil nanofluid was used in an all-glass evacuated solar absorber tubes without selective absorbing coating. For the ISAC, however, a coated evacuated collector tube of the same size filled with synthetic oil was used.

**Table 2:** Design parameters used for the theoretical calculations and the experimental setups.

Parameters	Values	Parameters	Values
Diameter of inner tube, *d*_1_	0.045 m	Absorption ratio of coating, α	0.86
Diameter of outer tube, *d*_2_	0.058 m	Transmittance of glass, τ_g_	0.93
Collector tube length, *L*	1.8 m	Dirt coefficient, τ_d_	0.97
Concentrator’s aperture width, *W*	1.04 m	Emissivity of glass, ε_g_	0.89
Optical efficiency factor, *f*	0.73	Emissivity of coating, ε_co_	0.09

To compare the steady state operating characteristics of the NDASC and the IASC, it was assumed that the following operational parameters remained unchanged with time: the global solar radiation intensity at 700 W/m^2^, ambient air temperature at 30 °C, ambient wind velocity at 2 m/s, and inlet velocity of fluid inside the tube at 0.0475 m/s. In addition, the heat conduction within the collector tube is neglected in the heat transfer calculation, as the conductive thermal resistance is much smaller than the other thermal resistances due to convection and heat radiation.

For both collectors, due to the light focal function of their parabolic concentrator, the reflected solar radiation intensity around the tube surface is extremely non-uniform. Using the Monte Carlo ray tracing method for a concentrating solar collector [21] and the structure parameters listed in Table 2 and Figure 1, the solar radiation intensity along the circumference of collector tubes, $I_{\text{0}}(\theta )$, can be evaluated. For example, when angle θ is in the range of ($-\frac{5\pi }{12}$, $\frac{5\pi }{12}$), $I_{\text{0}}(\theta )$ is calculated by:

(17) $$I_{\text{0}}(\theta )=2fG\frac{dx}{d_{1}\theta }=4fG\frac{p}{d_{1}}\frac{1-\cos\theta }{\sin^{2}\theta }$$

where *G* is the incident global solar radiation intensity; *p* is the focal length of the parabolic trough concentrator; *f* the optical efficiency factor; coordinate of arc angle θ = 0 represents the lowest position of the collector tube circumference.

**Figure 6:**
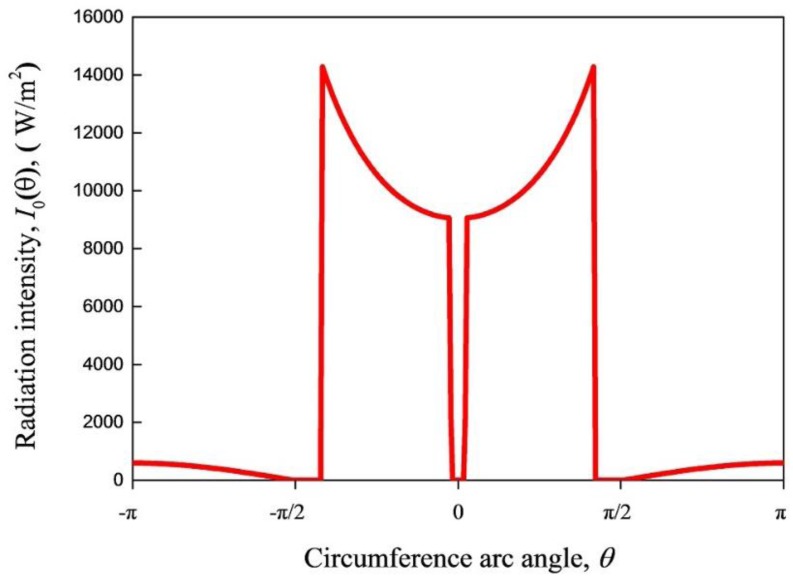
Solar radiation intensity along the circumference of collector tubes.

Figure 6 illustrates the solar radiation intensity along the circumference of collector tubes. It is suggested that reflected solar radiation intensity reached its highest when $\theta =\pm \frac{5\pi }{12}$.

#### 2.3.2. Temperature Distributions inside Collector Tubes

**Figure 7:**
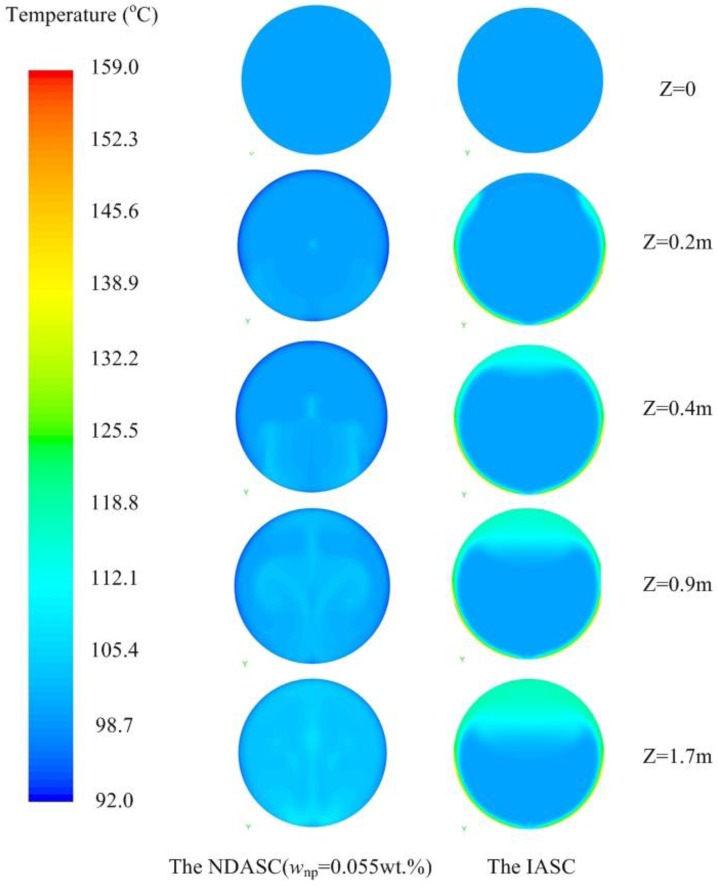
Fluid’s temperature distributions of cross sections at different length points (*t*_f,i_ = 100 °C).

The detailed temperature distributions of working fluid inside collector tubes for both the NDASC and the IASC were numerically evaluated and compared. Figure 7 shows the evaluated temperature distributions inside the collector tubes at a fluid’s inlet temperature (*t*_f,i_) of 100 °C. It can be seen that the internal fluid flow pattern and heat transfer caused by buoyancy force in the NDASC was obviously different from that in the IASC, and temperature distribution in the tube cross section in the NDASC was much more uniform than that in the IASC. For example, in the cross section near the outlet at tube length point *z* = 1.7 m, the inner tube wall temperature (*t*_w1_) in the IASC was about 35 °C higher than its internal fluid’s average temperature of 104 °C, with the maximum value of *t*_w1_ at 151 °C. By contrast, the inner tube wall temperature in the NDASC was about 5 °C lower than its internal fluid’s average temperature of 104.5 °C. This could be explained by the fact that solar radiation was totally absorbed on the wall surface for the IASC, while the solar radiation was transferred and absorbed by the nanofluid gradually layer by layer in the NDASC, resulting in a more efficient heat diffusion inside the nanofluid and thus a more uniform fluid’s temperature distribution in the NDASC. Also, the average fluid temperature at the outlet of the NDASC was higher, consequently leading to a higher overall solar collection efficiency.

**Figure 8:**
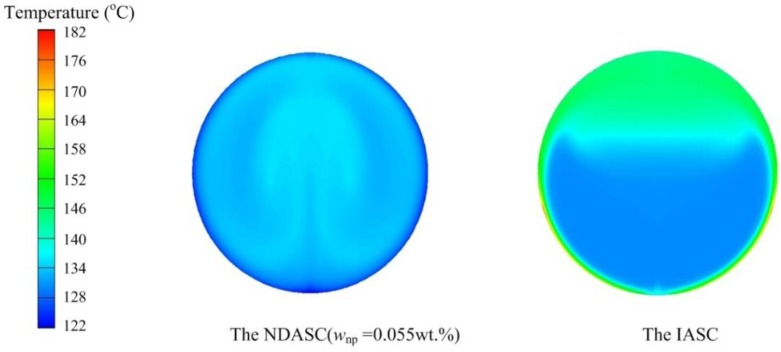
Fluid’s temperature distribution at *z* = 1.7 m in the NDASC and IASC (*t*_f,i_ = 130 °C).

Figure 8 shows the calculated fluid’s temperature distribution at *z* = 1.7 m in both the NDASC and the IASC, at a fluid’s inlet temperature of 130 °C. As seen, the average temperature of the nanofluid was even 11.5 °C higher than that of the tube wall near the outlet in the NDASC. By contrast, the maximum temperature in the IASC still occurred on the tube wall. Furthermore, in the IASC, its inner tube wall temperature was also extremely non-uniform, namely, the temperature in focal lines was much higher. The maximum temperature difference on the inner tube wall of the IASC was above 52 °C, which could increase the coating deformation and peeling, and cause collector tubes to be broken. However, the above problems were less serious in the NDASC.

#### 2.3.3 Solar Collection Efficiencies

**Figure 9:**
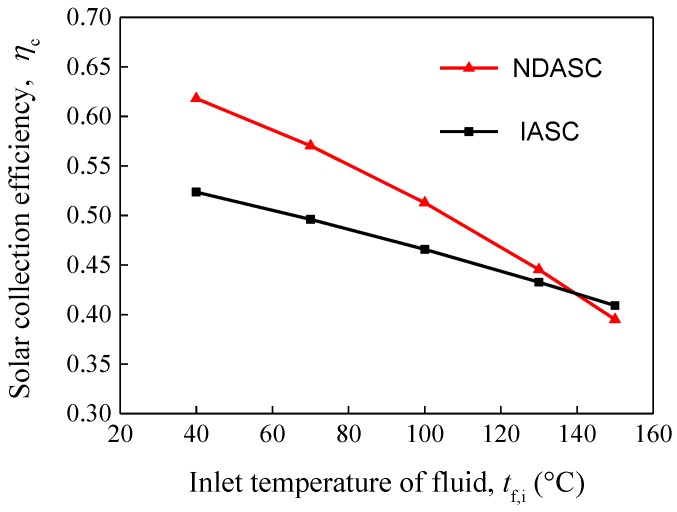
Variations of solar collection efficiencies with *t*_f,i_ for both the NDASC and the IASC.

The comparison of the evaluated solar collection efficiencies of the NDASC and the ISAC having a parabolic trough concentrator is shown in Figure 9. It can be found that solar collection efficiencies decreased with the inlet temperature of working fluid for both the NDASC and the ISAC. When the inlet temperature of fluid, *t*_f,i_, was lower than 139 °C, the solar collection efficiency of the NDASC was much higher than that of the ISAC. For example, when *t*_f,i_ was at 70 °C, η_c_ of the NDASC was close to 57% while that of the IASC was just about 49.6%. When *t*_f,i_ reached 130 °C, η_c_ of the NDASC was 44.5%, higher than that of the IASC at 43.3%. This was because the use of nanofluid in the NDASC can lead to a better performance in solar absorbing and heat transfer. However, it was obvious that the collection efficiency of the NDASC decreased sharply with *t*_f,i_, which was attributed to the much higher emissivity of the glass tube used in the NDASC than that of the selective absorption coating tube used in the IASC. According to Equation (7), given that the surface emissivity of the inner tube, ε_1_ was 0.89 for the NDASC but 0.09 for the IASC, the radiation heat loss of the NDASC, $q_{r1}$, would increase more significantly with an increase in *t*_w1_.

The solar collection efficiency curves for the two collectors suggested that the NDASC was superior to a conventional IASC within a preferred working temperature range, but inferior when the *t*_f,i_ exceeded a specific critical temperature (*t*_cr_). When *t*_f,i_ > *t*_cr_, the overall heat loss in the NDASC was larger, despite its lower wall temperature of the inner tube. In the current study, *t*_cr_ was at 139 °C. However, *t*_cr_ value may vary with the ambient conditions and solar collector’s structure.

## 3. Experimental Section

### 3.1. Experimental Setups for Solar Collection

**Figure 10:**
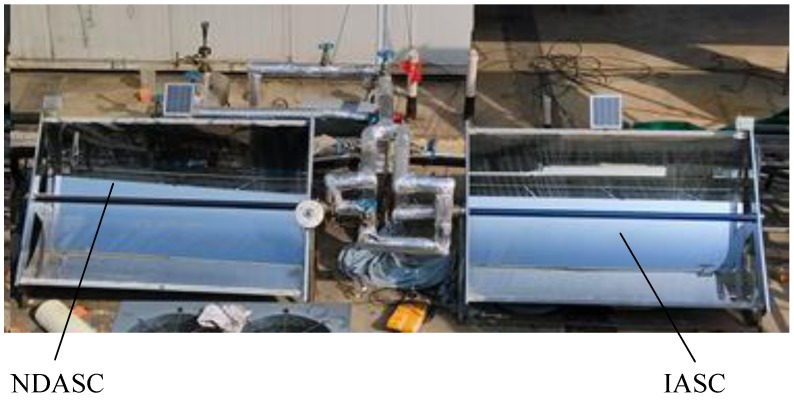
Photos of the experimental setups of the NDASC and the IASC.

The theoretical analysis in Section 2.3 suggested that the NDASC absorbed and transferred the solar heat to the working fluid more efficiently, and thus achieved higher solar collection efficiencies than that of the IASC within a preferred working temperature range. In order to further demonstrate the feasibility and superiority of the proposed NDASC, experimental setups of a NDASC and an ISAC using the same parabolic trough solar concentrator were established, as shown in Figure 10. The only differences between the two setups were the collector tubes and working fluid used. The design parameters for the experimental setups were the same with that used in the theoretical analysis, as shown in Table 2.

The reflecting mirrors of the solar concentrators in both setups were controlled to track the incident angle of solar radiation. The CuO/oil nanofluid and synthetic oil were continuously heated in the collector tubes of the NDASC and the IASC, respectively. For each collector tube, four PT100 temperature sensors (with an uncertainty of ±0.1 °C) were fixed inside the tube along the center axis and including the inlet point and outlet point, to measure the temperature of nanofluid and synthetic oil. A pyranometer (with a relative uncertainty of ±2%) was placed on the solar collector plane to measure the global solar radiation. At the same time, the temperature and humidity of ambient air were also measured. All the measurements were real-time computerized.

Using the obtained experimental data, the heat absorbed by the working fluid during a certain time interval (*t*_i_, *t*_i+1_), *Q*_f,i_, can be calculated by:

(18) $$Q_{\text{f,i}}=\int_{t_{i}}^{t_{i+1}}[w_{\text{np}}C_{\text{np}}+(1-w_{\text{np}})C_{\text{bf}}]m_{\text{nf}}dt_{\text{f}}$$

where *m*_nf_ is the mass of nanofluid, kg; *w*_np_ the mass fraction of nanoparticles; *C*_np_ and *C*_bf_ the specific heat of nanoparticles and base fluid (synthetic oil in this study), respectively, J/kg·K; *t*_f_ the fluid’s temperature, °C.

Although the experimental setups were operated under varied conditions during the entire experiment period, it could be reasonably assumed to be quasi steady-state operation in a specific time interval when the operating condition slightly varied. The solar collection efficiency of a concentrating solar collector, η_c,_ under a specific condition can therefore be derived by:

(19) $$\eta_{c}=\frac{Q_{\text{f,i}}}{WLG}$$

where, *W* and *L* are the aperture width and length of the solar concentrator, respectively, m; *G* the global solar radiation intensity, W/m^2^.

According to the uncertainties of measuring instruments used and the error transfer analysis, the relative error of the obtained experimental η_c_ was 8.4%.

### 3.2. Experimental Comparison of Solar Collection Efficiencies

**Figure 11:**
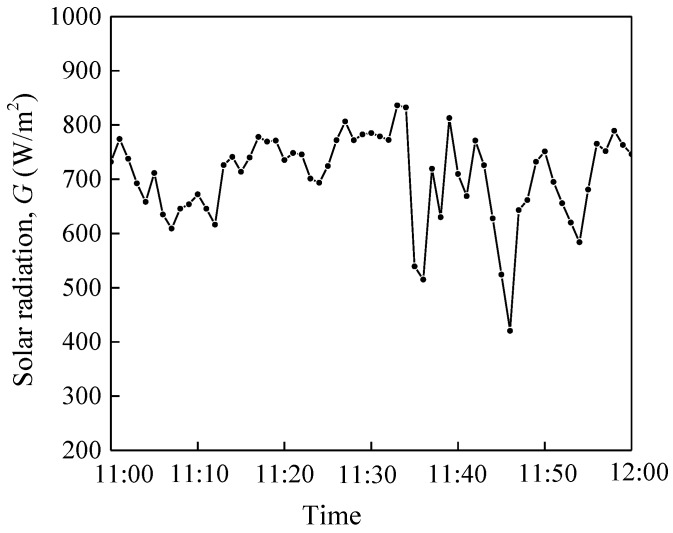
Variation of solar radiation with time during the experiment.

Experiments were carried out to compare the solar collection performances of the NDASC and the IASC setups described in Section 3.1. During a one hour experiment around noon (11:00 am to 12:00 pm), ambient air temperature varied between 29.0 and 30.6 °C, and the global solar radiation intensity on the titled surface of both collectors was between 500 and 850 W/m^2^ with an average value of 700 W/m^2^, as shown in Figure 11. The working fluid was heated from its initial temperature of 55 °C to above 160 °C.

**Figure 12:**
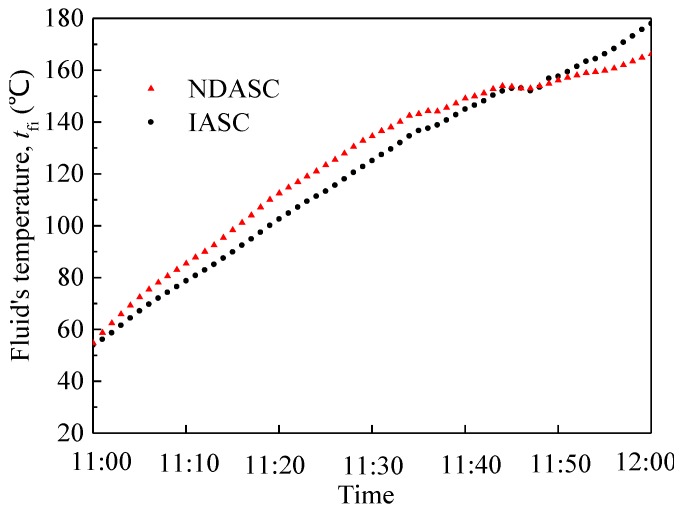
Variations of working fluid’s temperatures with time for two collectors.

Figure 12 shows the comparison between the temperature of CuO/oil nanofluid in the NDASC and that of synthetic oil in the conventional IASC during experiments. It can be seen that during the first 30 min, the temperature rise of nanofluid was faster than that of synthetic oil, demonstrating that the nanofluid absorbed solar radiation more quickly. However, when its temperature reached to above 130 °C, the increase in nanofluid temperature slowed down, while the increased rate of synthetic oil temperature remained unchanged, resulting in a lower temperature of the nanofluid than that of the synthetic oil.

**Figure 13:**
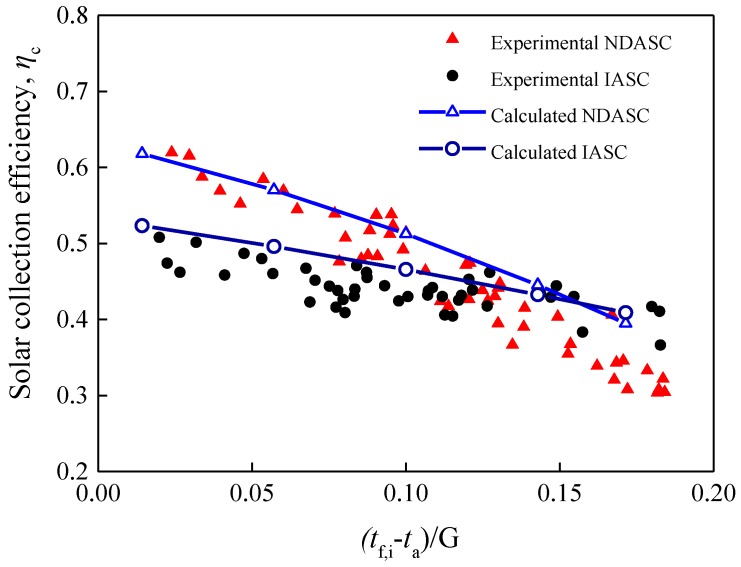
Comparison of the experimental and theoretical solar collection efficiencies for two collectors.

Using Equations (18) and (19), the experimental solar collection efficiencies for each time interval of 1 min were evaluated. As the solar radiation intensity varied with time during the experiment, the variations of η_c_ with (*t*_f,i_ − *t*_a_)/*G* were obtained and compared with theoretical values, as illustrated in Figure 13. Comparison results demonstrated that the experimental η_c_ decreased with (*t*_f,i_ − *t*_a_)/*G* for both the NDASC and the ISAC. Although the NDASC had a more remarkably decreasing trend in η_c_, it still achieved higher experimental solar collection efficiencies when (*t*_f,i_ − *t*_a_)/*G* was below 0.125. This agreed well with the variation trend of the theoretical η_c_, and verified the superiority of the proposed NDASC.

The experimental solar collection efficiencies were relatively lower than theoretically calculated ones for the following reasons. Firstly, the temperature sensors for measuring *t*_f,i_ were located along the center axis inside the tube, and hence there was a difference between the measured *t*_f,i_ and the actual average temperature of the working fluid. For the IASC, the measured *t*_f,i_ was lower than the average temperature at the cross section, causing the lower experimental values of η_c_. Secondly, the heat losses for the experimental setups were larger than that of the theoretically calculated ones, because there was additional heat loss at the tube inlet and outlet from the tube thermal insulation layer to ambient. As a result, the experimental value of the specific critical temperature (*t*_cr_) was 128 °C, lower than the theoretically calculated critical temperature of 139 °C presented in Section 2.3.3.

## 4. Conclusions

A novel NDASC with a parabolic trough concentrator and using nanofluid as working fluid was proposed for medium-temperature solar collection, and its operational characteristics were compared with a conventional IASC through both theoretical analysis and experiments. The main findings of this study are as follows:

- By adding nanoparticles, the solar absorptivity of a heat-transfer fluid can be significantly improved. Heat transfer in uncoated tube in the NDASC was more efficient than that in the IASC.
- The fluid’s temperature distribution in the tube cross section of the NDASC was much more uniform than that of the IASC. For a conventional IASC with concentrator, the maximum temperature always occurred on the tube wall, and the temperature in focal lines was much higher. By contrast, the temperature of the nanofluid inside uncoated tube was even higher than the tube wall temperature under certain condition for the NDASC.
- Theoretical and experimental results demonstrated that the proposed NDASC with a geometrical concentrating ratio of 7.36 obtained higher collection efficiencies than the IASC, when operated below a critical temperature value at 139 °C in theoretical analysis and 128 °C in experiments, respectively. The critical temperature may vary with the ambient conditions and solar collector’s structure.

The findings verified the feasibility of the proposed NDASC using nanofluid for solar-concentrating medium-temperature applications, so that an improved operational reliability and an enhanced solar collection efficiency within a preferred working temperature range can be expected.
